# Hydrogel composite scaffolds achieve recruitment and chondrogenesis in cartilage tissue engineering applications

**DOI:** 10.1186/s12951-021-01230-7

**Published:** 2022-01-06

**Authors:** Bo Huang, Pinxue Li, Mingxue Chen, Liqing Peng, Xujiang Luo, Guangzhao Tian, Hao Wang, Liping Wu, Qinyu Tian, Huo Li, Yu Yang, Shuangpeng Jiang, Zhen Yang, Kangkang Zha, Xiang Sui, Shuyun Liu, Quanyi Guo

**Affiliations:** 1 Institute of Orthopedics, Chinese PLA General Hospital, Beijing Key Lab of Regenerative Medicine in Orthopedics, Key Laboratory of Musculoskeletal Trauma & War Injuries PLA, No.28 Fuxing Road, Haidian District, Beijing, 100853 People’s Republic of China; 2 Department of Bone and Joint Surgery, The Affiliated Hospital of Southwest Medical University, No. 25 Taiping Road, Jiangyang District, Luzhou, 646000 Sichuan People’s Republic of China; 3 Department of Orthopaedics, Beijing Jishuitan Hospital, Beijing, 100035 China; 4 Hebei Medical University, Shijiazhuang, 050017 Hebei China

**Keywords:** Decellularized extracellular matrix, Peptide, Recruitment, Chondrogenesis, Cartilage regeneration

## Abstract

**Background:**

The regeneration and repair of articular cartilage remains a major challenge for clinicians and scientists due to the poor intrinsic healing of this tissue. Since cartilage injuries are often clinically irregular, tissue-engineered scaffolds that can be easily molded to fill cartilage defects of any shape that fit tightly into the host cartilage are needed.

**Method:**

In this study, bone marrow mesenchymal stem cell (BMSC) affinity peptide sequence PFSSTKT (PFS)-modified chondrocyte extracellular matrix (ECM) particles combined with GelMA hydrogel were constructed.

**Results:**

In vitro experiments showed that the pore size and porosity of the solid-supported composite scaffolds were appropriate and that the scaffolds provided a three-dimensional microenvironment supporting cell adhesion, proliferation and chondrogenic differentiation. In vitro experiments also showed that GelMA/ECM-PFS could regulate the migration of rabbit BMSCs. Two weeks after implantation in vivo, the GelMA/ECM-PFS functional scaffold system promoted the recruitment of endogenous mesenchymal stem cells from the defect site. GelMA/ECM-PFS achieved successful hyaline cartilage repair in rabbits in vivo, while the control treatment mostly resulted in fibrous tissue repair.

**Conclusion:**

This combination of endogenous cell recruitment and chondrogenesis is an ideal strategy for repairing irregular cartilage defects.

**Graphical Abstract:**

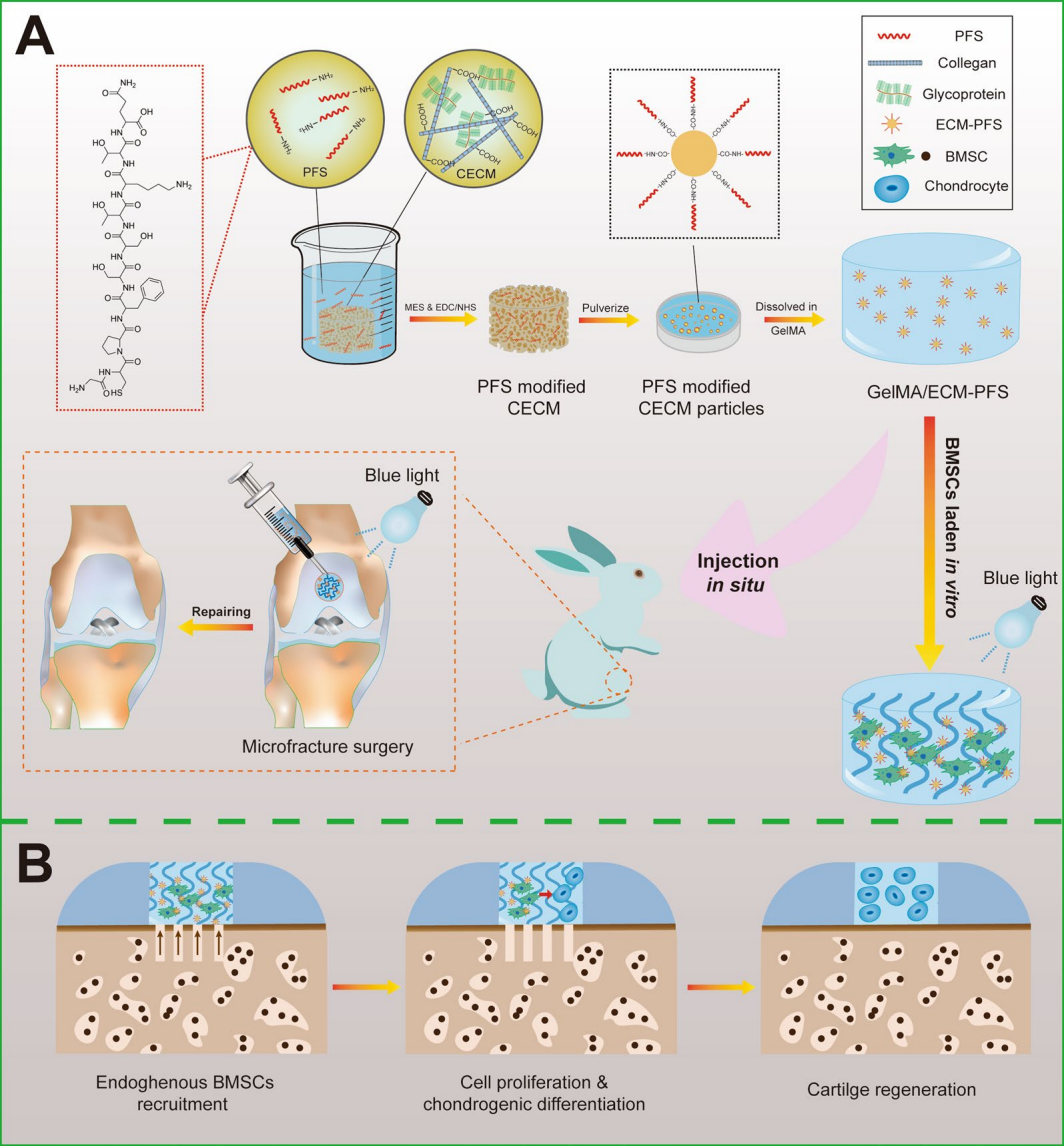

**Supplementary Information:**

The online version contains supplementary material available at 10.1186/s12951-021-01230-7.

## Introduction

Articular cartilage defects are common and often lead to gradual tissue degradation, joint pain, dysfunction and degenerative arthritis [1, 2]. However, as articular cartilage is avascular and aneural tissue with poor inherent repair ability, its regeneration is one of the most challenging clinical problems surgeons and scientists face [3, 4]. Cell-based tissue engineering techniques that promote cartilage self-repair with stem cell manipulation techniques, biological scaffolds, and biological factors have been extensively studied to address this challenge [5]. In recent years, a tissue regeneration approach using endogenous reparative cells at the site of in situ injury has been considered and used for articular cartilage repair [6]. The microfracture (MF) technique is commonly used to stimulate stem cells and/or progenitor cells in the bone marrow lumen to repair cartilage [7]. However, it has been reported that the levels of endogenous reparative cells are often insufficient after MF surgery due to low migration and local retention levels [8, 9]. Therefore, it is necessary to develop a cell-free scaffold with optimized structure and function to combine with MF to recruit more endogenous cells and achieve good cartilage regeneration. Moreover, since cartilage injuries are often clinically irregular [10, 11], tissue-engineered scaffolds that can be easily molded to fill cartilage defects of any shape and fit tightly into host cartilage are needed.

Gelatin methacrylate (GelMA) is a semisynthetic biomaterial prepared by chemically modifying gelatin and is widely used in cartilage tissue engineering [12]. GelMA combines the tunability of physical and chemical properties and the biological activity of gelatin [13]. Lithium acylphosphinate salt (LAP) initiates the system and improves the ease of the preparation process under blue light irradiation [14]. Although liquid biomaterial scaffolds have high formability for filling cartilage defects, their mechanical strength may be insufficient [15]. Extracellular matrix (ECM) extracted from cartilage is a natural biological material [16]. It has been reported that the mechanical properties of the GelMA hydrogel are significantly improved when ECM is incorporated [17]. In addition, ECM supports cell attachment and proliferation and promotes the formation of cartilage [16]. Moreover, in recent years, some peptide molecules have been found to regulate the migration and homing of mesenchymal stem cells [18–20]. The functional peptide sequence PFSSTKT (PFS) is a homing peptide discovered by phage display [18]. PFS is highly compatible with bone marrow mesenchymal stem cells (BMSCs) and promotes their differentiation [21].

**Fig. 1:**
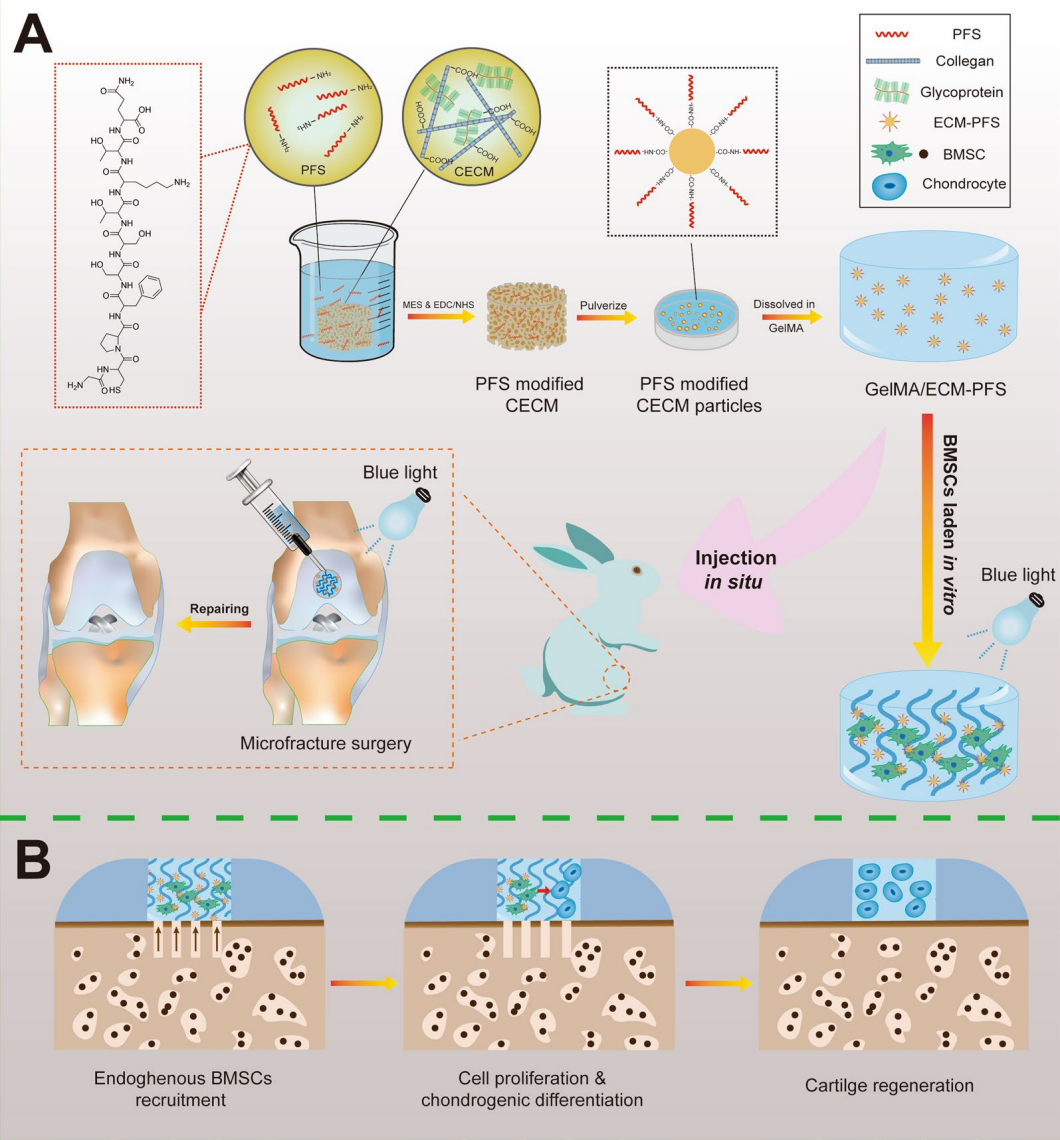
Schematic illustration of the overall study design. **A** Fabrication of peptide-functionalized composite hydrogel for knee cartilage regeneration in a rabbit chondral defect model. **B** The hyaline cartilage regeneration process facilitated by the peptide-functionalized composite hydrogel

Here, a composite hydrogel scaffold combining PFS-modified ECM (ECM-PFS) particles and GelMA hydrogel was designed to enhance MSC homing in a cartilage tissue engineering approach (Fig. 1). In this GelMA/ECM-PFS hydrogel scaffold, ECM-PFS particles improved the biomechanical properties of the scaffold, creating a microenvironment for chondrogenic differentiation and BMSC recruitment. Additionally, GelMA provided a favorable three-dimensional cell-supporting microenvironment that could bind tightly to host cartilage and fill defects.

To the best of our knowledge, this is the first attempt to apply a GelMA/ECM-PFS hydrogel in cartilage tissue engineering. We hypothesize that the GelMA/ECM-PFS hydrogel combined with MF treatment will improve the quality of the regenerated cartilage tissue. Our functional bone marrow-specific scaffolding system provides new insights for an efficient strategy for clinical articular cartilage repair and represents a promising therapeutic for articular cartilage regeneration in the future.

## Materials and methods

### Preparation and construction of GE hydrogel

The preparation of GelMA was based on a previous method with minor modifications [12, 13], and the degree of substitution (DS) of GelMA was determined by 1H-NMR spectra. ECM was prepared from fresh porcine articular cartilage tissue using physicochemical methods with some modifications[16]. To construct a GE composite hydrogel, four different concentrations (0, 1, 1.5 and 2% w/v) of ECM were added to the GelMA solution (10%, w/v), and the concentration of LAP photoinitiator was 0.25% w/v. The composite hydrogel GE was formed by exposure to blue light (405 nm) irradiation for 2 min in a cylindrical mold with a diameter of 1 cm. To determine the ECM concentration with the best mechanical properties for use in the GE composite hydrogels, we calculated the compression modulus of each sample with an Instron 5969 mechanical analyzer. Detailed experimental procedures are available in Additional file 1: Sections S1.1 and S1.2.

### Construction of GelMA/ECM-PFS

To increase the adhesion and homing ability of BMSCs in ECM and the ability of composite hydrogel to recruit endogenous stem cells, we designed and prepared PFS-functionalized ECM and added it to a GelMA hydrogel. In brief, ECM was lyophilized, and PFS was combined with ECM after activation by MES, EDC and NHS. Imaging of rhodamine-labeled PFS (RhoB-PFS)-functionalized ECM sponges was performed using Nikon confocal microscopy to evaluate PFS distribution in ECM sponges. The particle size distribution of PFS-ECM particles was analyzed by dynamic light scattering after pulverizing the PFS-ECM composite scaffold. Finally, an ECM composite hydrogel consisting of PFS-ECM, GelMA and LAP was prepared, transferred to the same cylindrical mold and exposed to blue light to prepare a PFS-functionalized hydrogel. Detailed experimental procedures are available in Additional file 1: Section S1.3.

### Characterization of composite hydrogel

The morphology of the hydrogels was visualized using a stereomicroscope, and the microstructure of the lyophilized hydrogels was further characterized by scanning electron microscopy (SEM). Then, pore sizes in the lyophilized hydrogels were observed and calculated by ImageJ software. The porosity of the scaffolds was measured using an ethanol displacement method. Finally, we determined the enzymatic degradation and swelling properties of different hydrogels. Detailed experimental procedures are available in Additional file 1: Section S1.4.

### Cell isolation, culture and identification

BMSCs were extracted from rabbit bone marrow using Percoll density gradient centrifugation [22], as previously reported. In brief, bone marrow blood was extracted from a 1-month-old New Zealand rabbit by puncture with a bone-piercing needle. A lymphocyte separator was added to separate the mixture into four layers. The second layer, containing mesenchymal stem cells, was transferred to a new centrifuge tube, diluted with PBS, washed and centrifuged. Then, the cells were suspended in medium and transferred to a culture flask. We proved the adipogenic, osteogenic and chondrogenic differentiation potential of the BMSCs through a trilineage-induced differentiation experiment. Moreover, we detected the positive and negative expression of surface markers of BMSCs by flow cytometry. Detailed experimental procedures are available in Additional file 1: Section S1.5.

### In vitro cytocompatibility study

The cytocompatibility of the hydrogels was evaluated in detail. To assess the viability of BMSCs in different hydrogels, a Live/Dead Assay Kit was used. Cell Counting Kit-8 was used to quantify the proliferation of BMSCs in different hydrogels [23]. The morphology of BMSCs in GelMA, GelMA/ECM and GelMA/ECM-PFS hydrogels was assessed using DAPI staining and FITC- phalloidin staining after 1 d and 7 d of culture, according to the manufacturer’s instructions. Detailed experimental procedures are available in Additional file 1: Section S1.6.

### In vitro and in vivo MSC recruitment

A Transwell system was used to determine the BMSC recruitment ability of composite hydrogels in vitro [24]. We established a rat full-thickness cartilage defect model to investigate the ability of GelMA, GelMA/ECM and GelMA/ECM-PFS hydrogels combined with MF surgery to enhance BMSC migration in vivo. Detailed experimental procedures are available in Additional file 1: Section S1.7.

### In vitro chondrogenic differentiation

To explore whether the incorporation of ECM and ECM-PFS can promote the chondrogenic differentiation of BMSCs in the GelMA hydrogel, we cultured the three hydrogels with BMSCs in chondrogenic differentiation medium to construct tissue-engineered cartilage in vitro. Tissue-engineered cartilage samples were collected after 2 weeks of culture, fixed with paraformaldehyde, dehydrated, embedded in paraffin, and cut into 6 μm sections. Immunohistochemical staining was used to evaluate type II collagen in tissue-engineered cartilage. Finally, the glycosaminoglycan (GAG) and hydroxyproline (HYP) contents of tissue-engineered cartilage were determined by the Hydroxyproline Assay Kit and the Tissue Total GAG Content DMMB Colorimetry Kit as per the experimental protocol. Detailed experimental procedures are available in Additional file 1: Section S1.8.

### In vivo animal studies

Animal experiments were approved by the Institutional Animal Care and Use Committee at the PLA General Hospital. Thirty-two male New Zealand rabbits (6 months old, 2.5–3.0 kg, n = 8 knees per group) were randomly divided into 4 groups, the microfracture (MF), microfracture combined with GelMA/ECM implantation (GelMA/ECM), microfracture combined with GelMA/ECM-PFS implantation (GelMA/ECM-PFS) and sham groups. Euthanasia was performed 3 months and 6 months after surgery, after which the knee joints were collected for macroscopic evaluation and micro-CT scanning. To reproduce the biomechanical properties of the tissue, we tested the compression modulus of each group of samples after micro-CT scanning. After micro-CT scanning, hematoxylin and eosin (H&E), Safranin-O, toluidine blue, and Sirius red staining were used to evaluate the morphology and arrangement of the neotissue and identify GAGs. Immunohistochemical staining was used to evaluate the secretion of type II collagen in regenerated tissues. After staining, the regenerated tissue was evaluated according to the ICRS histological scoring system. Moreover, the HYP and GAG contents of the regenerated tissue were determined by the Hydroxyproline Assay Kit and the Tissue Total GAG Content DMMB Colorimetry Kit according to the manufacturer’s instructions. Detailed experimental procedures are available in Additional file 1: Section S1.9.

### Statistical analysis

All statistical analyses were performed using SPSS V.20.0 (IBM; Armonk, New York, USA). For normally distributed data, one-way analysis of variance (ANOVA) or Student’s t-test was used for quantitative data; otherwise, the nonparametric Kruskal–Wallis test was used. Statistical significance was set at a two-sided p-value of < 0.05.

## Results

### Preparation and characterization of three hydrogels

**Fig. 2:**
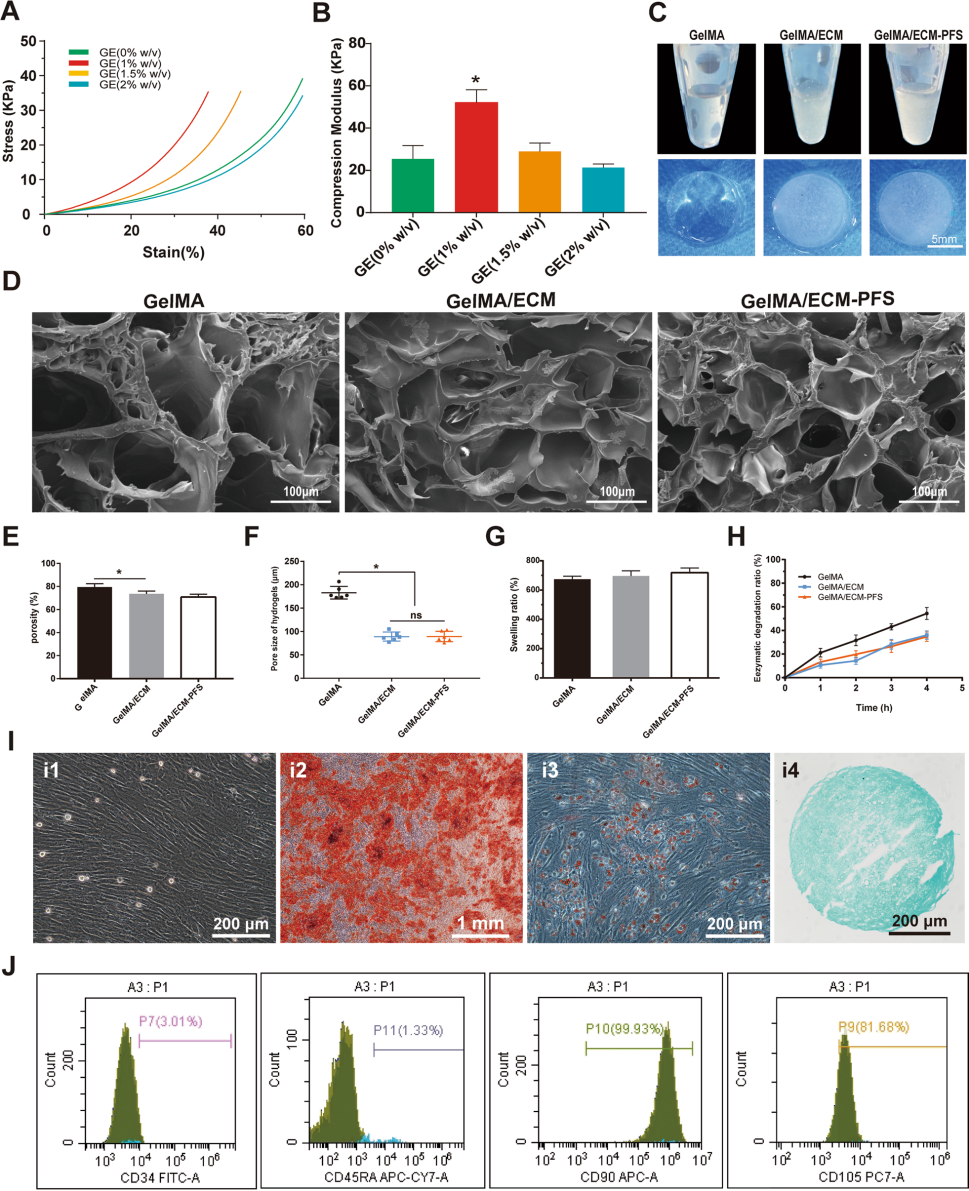
Fabrication and characterization of hydrogels and the culture and identification of BMSCs. **A** Stress–strain curve of composite hydrogels with different CECM concentrations (0, 1, 1.5, 2) w/v. **B** Compression modulus of composite hydrogels. **C** Gross observation of the three different hydrogels (GelMA, GE, GelMA/ECM-PFS). **D** SEM images of the three different hydrogels. **E**, **F** Porosity and pore size of the three lyophilized hydrogels. **G** The swelling ratio of the three lyophilized hydrogels when no longer water-absorbing. **H** The degradation rate of the three lyophilized hydrogels in type II collagenase solution. **I** Culture of BMSCs (i1), osteogenic (i2), adipogenic (i3) and chondrogenic (i4) differentiation of BMSCs. **J** Flow cytometry of surface proteins. Data are the means ± SD (*p < 0.05, ns represents no significant difference, n = 3)

We successfully synthesized a GelMA hydrogel, and the hydrogen spectrum analysis showed that the grafts were attached. The degree of substitution reached approximately 75% after adding MA (Additional file 1: Fig. S1). The chemical cross-linking agent between the amino group in PFS and the carboxyl group in DCECM was synthesized into PFS-functionalized ECM by MES and EDC/NHS activation. The results shown in Additional file 1: Fig. S2 demonstrate that Rho-B-labeled PFS was uniformly coupled to ECM sponges. Subsequently, the PFS-functionalized ECM sponge was dissected, dissolved in deionized water, and subsequently exposed to ultrasound using a Q125 Sonicator (Qsonica, USA) to generate homogeneous particles (Additional file 1: Fig. S3). Before preparing the GelMA/ECM-PFS hydrogel, we explored the influence of ECM particles at different concentrations on the mechanical properties of the composite hydrogel. The compression modulus of the hydrogels was as follows: pure GelMA hydrogel, 25.43 ± 6.33 kPa; GelMA with 1% ECM, 52.07 ± 6.12 kPa; GelMA with 1.5% ECM, 28.93 ± 4.05 kPa; and GelMA with 2% ECM, 21.30 ± 1.76 kPa. GelMA with 1% (w/v) concentration of ECM was selected for subsequent experiments (Fig. 2A, B).

As shown in Fig. 2C, the pure GelMA hydrogel precursor solution was colorless and transparent and became white and homogeneous after adding ECM and ECM-PFS particles. We exposed pure GelMA solution, GelMA/ECM solution and GelMA/ECM-PFS solution to blue light. After irradiation, stable cylindrical hydrogels formed in the mold. SEM was used to characterize the microstructures of the three hydrogels. As shown in Fig. 2D, the three groups of hydrogels showed loose and porous structures after freeze-drying, and the pore sizes (Fig. 2E) of the GelMA/ECM group and GelMA/ECM-PFS group were 89.11 ± 9.69 μm and 89.35 ± 11.01 μm, respectively, slightly lower than those of the GelMA group (182.90 ± 13.45 μm) (p < 0.05). There was no significant difference in pore size between GelMA/ECM and GelMA/ECM-PFS. In addition, the porosities of the three groups of hydrogels were measured and found to be 79.45 ± 2.92% for the GelMA hydrogel, 73.65 ± 2.28% for the GelMA/ECM hydrogel and 70.80 ± 2.49% for the GelMA/ECM-PFS hydrogel (Fig. 2F). We also tested the swelling properties of the hydrogels. The results showed that the swelling rates were 674.5 ± 20.04% (GelMA), 696.4 ± 35.02% (GelMA/ECM) and 718.8 ± 31.94% (GelMA/ECM-PFS), and there were no significant differences among the three groups (Fig. 2G). Enzymatic degradation tests showed that GelMA/ECM and GelMA/ECM-PFS hydrogels degraded at similar rates throughout the incubation period. The degradation rate of the GelMA group was faster than that of the GelMA/ECM and GelMA/ECM-PFS groups after 2 h (Fig. 2H). These results indicated that the three hydrogels had internal porous three-dimensional structures, high porosity levels and good swelling properties.

### Culture and identification of BMSCs

BMSCs were isolated and cultured and demonstrated multidirectional differentiation ability (Fig. 2I). The cultured BMSCs showed a uniform, slender, spindle shape under the microscope (Fig. 2i1). After 14 days of culture in osteogenic induction medium, Alizarin red staining showed a large amount of matrix calcification with calcium nodule formation (Fig. 2i2), indicating that the BMSCs were capable of osteogenic differentiation. After 7 days of culture in adipogenic induction medium, a large number of rounds, red-stained lipid droplets were observed with Oil red-O staining (Fig. 2i3). After 21 days of culture in chondrogenic induction medium, Alcian blue staining was positive (Fig. 2i4), indicating that the 3D cell pellets were rich in proteoglycans. The above experimental results confirm that the BMSCs are capable of multilineage differentiation.

The flow cytometry results (Fig. 2J) of BMSCs showed that the cells expressed the MSC surface markers CD90 (99.93%) and CD105 (81.68%) and did not express the hematopoietic cell markers CD34 (3.01%) and CD45RA (1.33%). These data indicated that the isolated BMSCs had good homogeneity, without hematopoietic or endothelial cells, and expressed MSC characteristics.

### Cytocompatibility of hydrogels

**Fig. 3:**
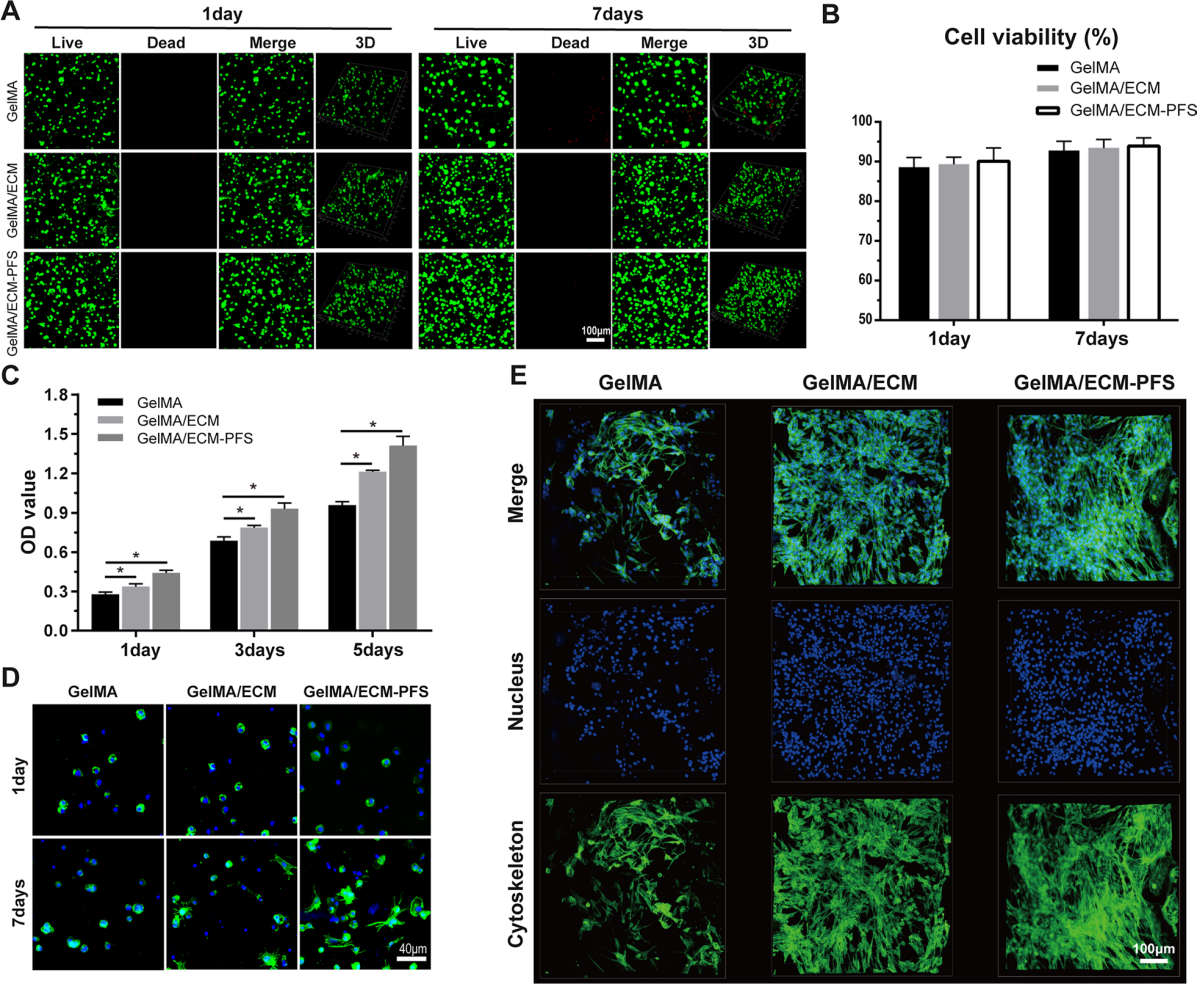
Cytocompatibility of hydrogels and cellular morphology of BMSCs in different hydrogels. **A**, **B** Live/dead staining and viability of BMSCs cultured in different hydrogel scaffolds for 1 and 7 days. **C** CCK-8 OD value of different hydrogels cultured with BMSCs. **D** FITC-cytoskeleton staining of BMSCs cultured in different hydrogels for 1 and 7 days. **E** The morphology of BMSCs on different hydrogel scaffolds for 7 days. Data are the means ± SD (*p < 0.05, ns represents no significant difference, n = 3)

The cytocompatibility of the hydrogels was evaluated by cell viability, cell proliferation, and cell adhesion tests. Live/dead staining showed that BMSCs grew evenly in the GelMA, GelMA/ECM and GelMA/ECM-PFS scaffolds. In all three hydrogels, living cells were arranged mainly along the orthogonal printed fibers. A few cells were stained fluorescent red (dead cells). We calculated the cell viability in each group through quantitative analysis, and the results showed that all three hydrogels yielded good cell viability, above 85% on day 1 and 90% on day 7, indicating that all scaffolds supported cell growth (Fig. 3A, B).

We then quantitatively measured the proliferation of BMSCs in various hydrogels by CCK-8 assay. As shown in Fig. 3C, the OD values of cells cultured in all hydrogels increased over time, and the absorbance values of the GelMA/ECM and GelMA/ECM-PFS groups on days 1, 3 and 7 were significantly higher than those of the GelMA group. These results indicate that ECM-containing hydrogels induced stronger cell proliferation, and the addition of PFS enhanced the ability of the hydrogel to promote BMSC proliferation.

Cell adhesion and growth morphology were further observed by fluorescence confocal scanning microscopy after 1 and 7 days of seeding. Figure 3D shows cytoskeletal protein F-actin (green) and nuclei (blue) staining of cultured BMSCs in the hydrogels [25]. On day 1 after cell inoculation, BMSCs in the three hydrogels were scattered and maintained a spherical shape. Seven days after inoculation, the BMSCs in the GelMA hydrogel were still spherical, while the cells in the GelMA/ECM hydrogel were spindle-shaped. More cells in the GelMA/ECM-PFS hydrogel showed spindle morphology and pseudopodia than cells in the GelMA/ECM hydrogel, and the cell diffusion area was larger. To further explore the affinity of hydrogels to BMSCs in each group, BMSCs were inoculated on the surface of aforementioned cell-free hydrogels (Fig. 3E). After 7 days of culture, BMSCs on GelMA and GelMA/ECM were spherical in shape, while cells on GelMA/ECM-PFS hydrogels were almost fully extended and had a larger surface area. These results further confirm that including ECM-PFS in hydrogel scaffolds is beneficial for cell adhesion and proliferation.

### In vitro and in vivo MSC recruitment

**Fig. 4:**
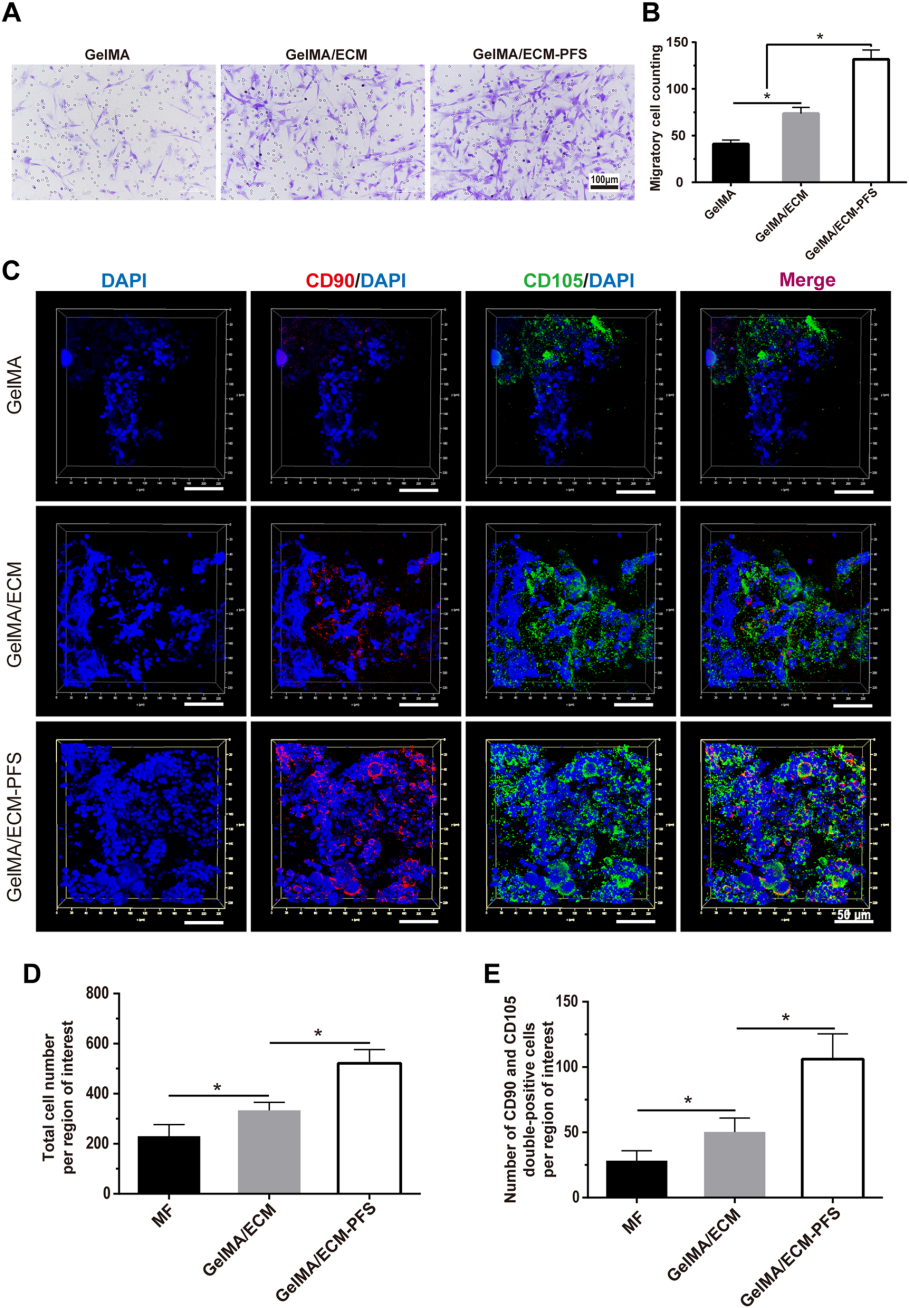
In vitro and in vivo recruitment of stem cells by the different hydrogels. **A**, **B** Crystal violet staining of the migrated BMSCs in the GelMA group, GE group and GelMA/ECM-PFS group in vitro, plus a statistical analysis. **C** Confocal images of endogenous stem cell migration assay in vivo. **D** The total number of cells recruited into the cartilage defect site. **E** The number of CD90 and CD105 double-positive cells recruited into the cartilage defect site. Data are the means ± SD (*p < 0.05, n = 3)

We performed a Transwell assay to verify BMSC migration toward different hydrogels in vitro (Fig. 4A). After adding extracts of the three hydrogel groups to the lower chamber, migrated BMSC numbers after 12 h were significantly higher in the GelMA/ECM group than in the GelMA group (Fig. 4A, B), and BMSC migration in the GelMA/ECM-PFS group was significantly higher than that in the GelMA/ECM group. These results indicated that both the GelMA/ECM and GelMA/ECM-PFS hydrogels could promote BMSC migration and that this effect was optimal with GelMA/ECM-PFS hydrogels.

The recruitment ability of different hydrogels in vivo was also demonstrated. Two weeks after implantation in vivo, a higher total cell number was observed with GelMA/ECM and GelMA/ECM-PFS hydrogels, and the cell distribution was richer and more uniform (Fig. 4C, D). Moreover, the number of CD90 and CD105 double-positive cells in the GelMA/ECM-PFS hydrogel group was significantly increased compared with that in the other two groups (Fig. 4C, E). Together, these results suggested that ECM components could effectively enhance the ability of the hydrogel system to recruit peripheral MSCs to cartilage defects, while ECM-PFS in the composite hydrogel enhanced the recruitment ability.

### Chondrogenic differentiation of BMSCs in hydrogels in vitro

**Fig. 5:**
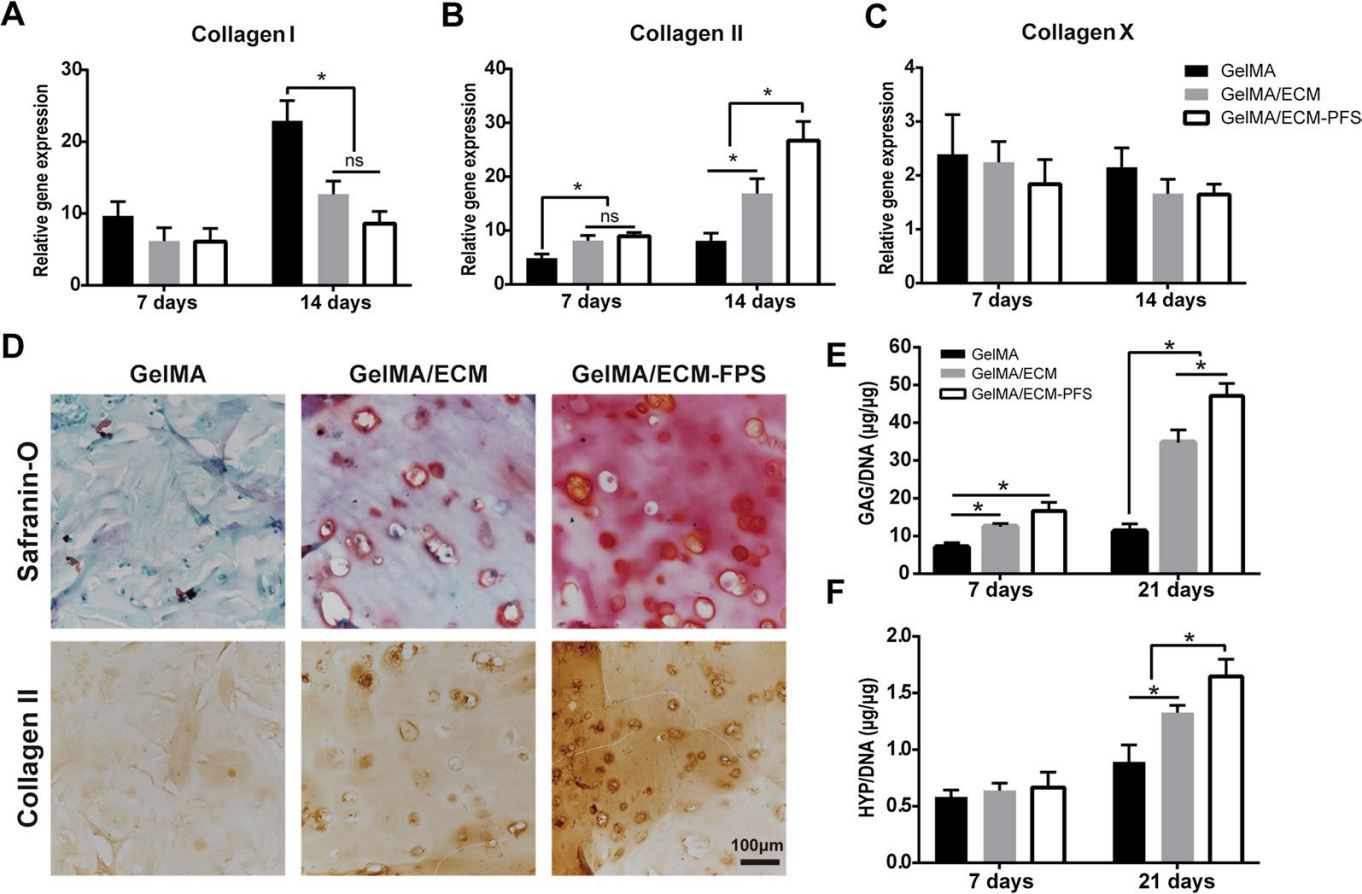
In vitro chondrogenic assays. **A**–**C** Expression of genes related to chondrogenic differentiation of BMSCs cultured in the different hydrogels. **D** Pathological staining of cartilage matrix components (Safranin-O staining for GAG, immunohistochemical staining of type II collagen). **E**, **F** Quantitative detection of GAG and HYP (collagen) after culturing for 7 and 21 days. Data are the means ± SD (*p < 0.05, ns represents no significant difference, n = 3)

To investigate the biological activity of the three groups of hydrogels on chondrogenic differentiation of BMSCs in vitro, tissue-engineered cartilage was constructed and assessed for chondrogenesis using RT-qPCR, histological and immunohistochemical staining, and biochemical analysis (Fig. 5). As shown in Fig. 5B, the gene expression level of Collagen II in the GelMA/ECM and GelMA/ECM-PFS groups was significantly higher than that in the GelMA group after 7 days of hydrogel culture, and the gene expression level was the highest in the GelMA/ECM-PFS group after 14 days. Collagen I and Collagen X were expressed at low levels in GelMA/ECM-PFS hydrogels (Fig. 5A, C).

We used Safranin-O and immunohistochemical staining of collagen II to evaluate the cartilage matrix generated by BMSCs in hydrogels (Fig. 5D). Safranin-O staining, associated with proteoglycan synthesis, showed proteoglycan production was optimal in GelMA/ECM-PFS. In addition, immunohistochemical staining for collagen II confirmed that ECM-PFS significantly promoted the deposition of collagen II. Meanwhile, the content of GAG and HYP in the tissue-engineered cartilage constructed by the GelMA/ECM-PFS group was highest after 21 days of culture (Fig. 5E, F).

Taken together, these results suggest that ECM-containing hydrogels can provide a microenvironment conducive to the differentiation of BMSCs into chondrocytes and that modification of the PFS peptide enhances BMSC differentiation in hydrogels.

### Cartilage regeneration in vivo

#### Macroscopic evaluation and biomechanical analysis

**Fig. 6:**
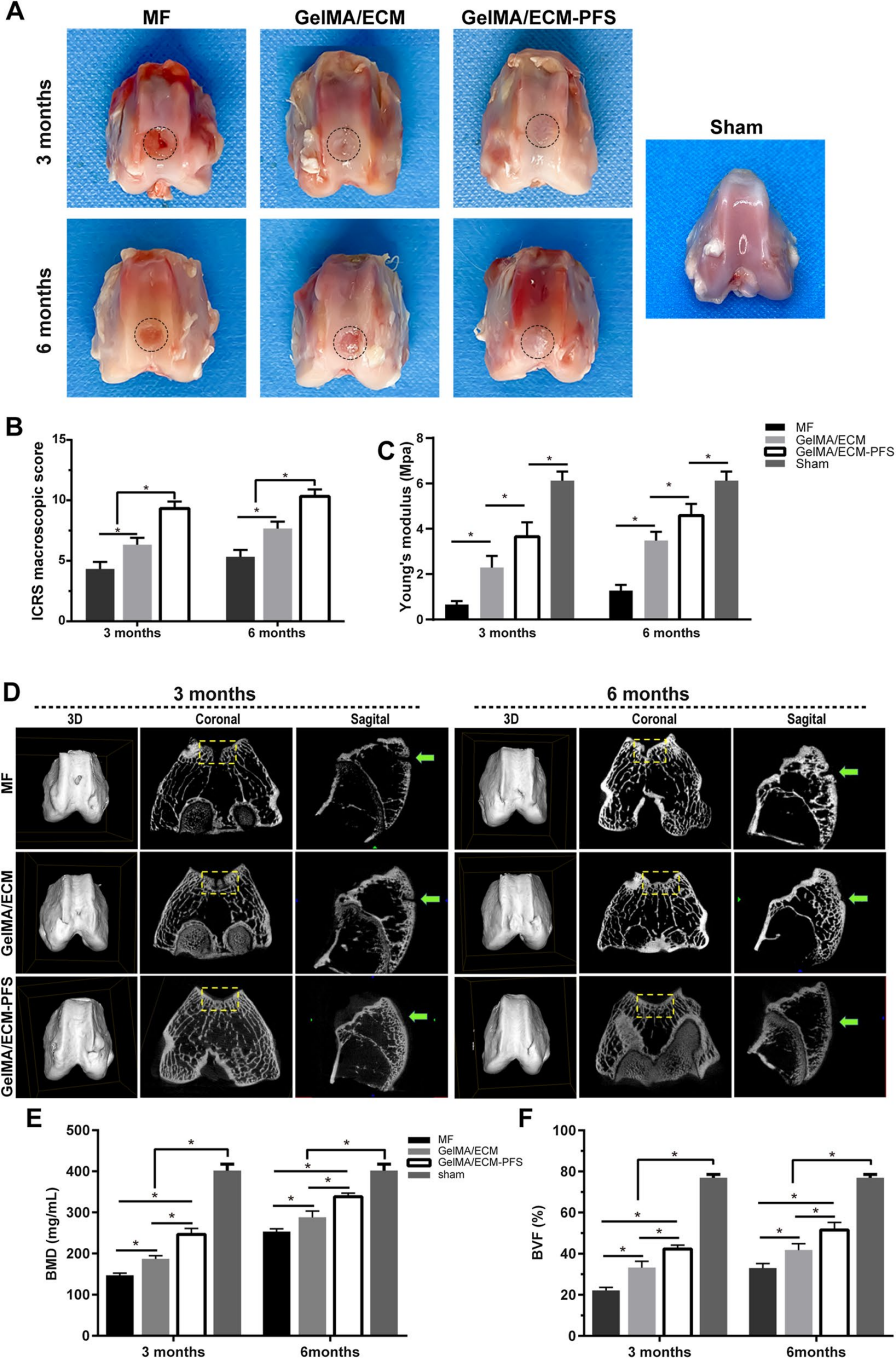
Macroscopic evaluation, biomechanical properties and micro-CT evaluation of repaired knees. **A** Representative macroscopy of repaired tissues at 3 and 6 months postoperation. Blue circles indicate the defect area. **B** International Cartilage Research Society (ICRS) score for macroscopic assessment (n = 8 knees for each time point). **C** Young's modulus (n = 4 knees). **D** Micro-CT images showing 2D and 3D reconstruction of the repaired cartilage at 3 and 6 months after surgery. Yellow squares and green arrows indicate the defect area. Quantitative analysis of **E** BMD and **F** BVF in the defect area. Data are the means ± SD (*p < 0.05, n = 4)

Thirty-two male rabbits (6 months old, 2.5–3 kg, n = 8 knee joints per group) were selected for the experimental animal model. The rabbits were divided into 4 groups for treatment with the 3 hydrogel scaffolds and the control to evaluate the ability of each treatment to repair knee cartilage. As shown in Fig. 6A, 3 months after repair, the defect area in the MF group was partially filled by the repaired tissue, with some defects remaining. There was a clear boundary between the edge of the repaired tissue and the surrounding normal articular cartilage. In the GelMA/ECM group, the osteochondral defect area was filled with new tissue, and the repaired surface was rough. The dark red repaired tissue was not well integrated with the surrounding normal articular cartilage, and there were still clear boundaries. However, in the GelMA/ECM-PFS group, the osteochondral defect area was well filled with new tissue, the repair plane was smoother and flat, and the degree of fusion with the surrounding normal articular cartilage was higher. Until 6 months after repair, the osteochondral defect area in the MF group was filled with repaired tissue, but the surface remained rough, and the boundary with the surrounding normal articular cartilage was obvious. The new tissue in the defect area of the GelMA/ECM group showed a significant improvement in fusion compared with that at 3 months postoperatively. However, the visible boundary between the pale and uneven repair plane and the surrounding normal articular cartilage remained. The osteochondral defect area in the GelMA/ECM-PFS group was filled with cartilage-like tissue, the surface was flat and smooth, well fused with the surrounding normal cartilage tissue, without clear boundaries, and the repaired cartilage appeared close to the cartilage of the sham group.

The ICRS score of the GelMA/ECM-PFS group (3 months: 9.33 ± 0.57; 6 months: 10.33 ± 0.58) was significantly higher (p < 0.05) than that of the other two groups. At both time points, the ICRS score of the GE group (3 months: 6.33 ± 0.58; 6 months: 7.67 ± 0.58) was also significantly higher (p < 0.05) than that of the MF group (3 months: 4.25 ± 0.50; 6 months: 5.33 ± 0.58), as shown in Fig. 6B.

The biomechanics evaluation (Fig. 6B) showed that the neocartilage in the GelMA/ECM-PFS group was more similar to normal cartilage (6.13 ± 0.23 MPa) than that in the GE group (GelMA/ECM-PFS group: 3.65 ± 0.37 MPa (3 months), 4.59 ± 0.29 MPa (6 months); MF: 0.66 ± 0.09 MPa (3 months), 1.27 ± 0.15 MPa (6 months); GE: 2.29 ± 0.29 MPa (3 months), 3.48 ± 0.22 MPa (6 months)).

#### Micro-CT analysis

In the micro-CT reconstructed images at different time points in each group (Fig. 6D), the yellow boxes and green arrows indicate cartilage defects in the coronal and sagittal planes, respectively. These images revealed that the defects in the GelMA/ECM-PFS group were almost filled with well-integrated newly formed bone and cartilage-like tissue, and the cartilage layer was smooth and continuous at both 3 and 6 months after surgery. However, the other two groups still had obvious unrepaired blank areas and broken cartilage layers, even after 6 months. The level of bone regeneration was observed to be higher than that in the other two control groups (Fig. 6D).

Micro-CT quantitative analyses of bone mineral density (BMD) and bone volume fraction (BVF) confirmed this result. As shown in Fig. 6E, F, whether 3 months or 6 months after surgery, the main evaluation indicators of subchondral bone reconstruction in the GelMA/ECM-PFS group, BMD (3 months: 246.40 ± 14.45 mg/cm3; 6 months: 338.3 ± 8.68 mg/cm^3^) and BVF (3 months: 38.82 ± 3.01%; 6 months: 44.85 ± 2.18%), were significantly better (p < 0.05) than those in the other two groups. At 3 months after surgery, BMD (187.00 ± 7.89 mg/cm^3^) and BVF (37.85 ± 1.93%) in the GelMA/ECM group were significantly better than (p < 0.05) those in the MF group (BMD: 146.70 ± 5.66 mg/cm^3^; BVF: 27.75 ± 4.88%). At 6 months after surgery, the performance of the GE group (BMD: 288 ± 15.11 mg/cm^3^; BVF: 39.44 ± 1.44%) was still slightly better than that of the MF group (BMD: 253.30 ± 6.75 mg/cm^3^; BVF: 37.33 ± 1.92%).

In conclusion, the micro-CT results prove that ECM-PFS functionalized hydrogel has the best ability among the three tested hydrogels to repair cartilage, especially in the first three months after surgery. However, there was still a significant difference between the reconstructed subchondral bone and the subchondral bone of the sham group (Fig. 6E, F).

#### Histological and IHC staining

**Fig. 7:**
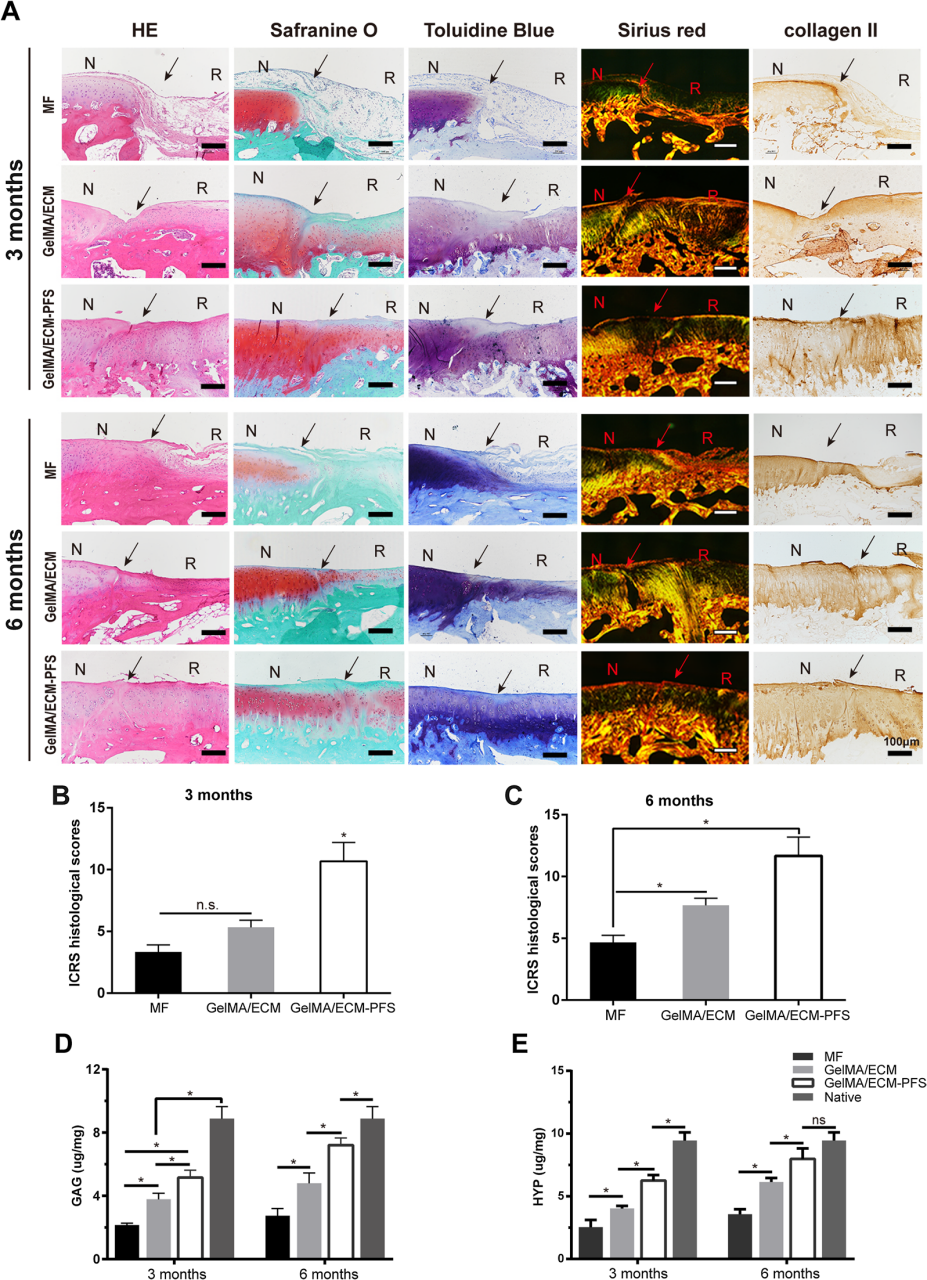
Histological, immunohistochemical, biochemical, and biomechanical evaluation of repaired tissue. **A** Histological (H&E, Safranin-O, toluidine blue and Sirius red staining) and immunohistochemical analyses of the defect area. Black solid arrows indicate the repair interface (N: normal cartilage; R: repaired cartilage). The ICRS histological scores for histological evaluation of cartilage repair after 3 months (**B**) and 6 months (**C**). The total cartilage-specific matrix content **D** GAG and **E** HYP indicated collagen. Data are means ± SD (*p < 0.05, n.s. represents no significant difference)

Three months after the surgery, H&E staining showed disordered regenerated tissue in the MF group (Fig. 7A), and only a few cells were present. The level of regenerated tissue and the number of regenerated cells in the GelMA/ECM group was greater than that in the MF group, but the tissues and cells were still poorly arranged. In contrast, in the GelMA/ECM-PFS group, the more organized chondrocyte-like cells there were, the better the integration of the repaired tissue with the surrounding normal tissue was; however, the regenerated tissue was slightly thinner. The Safranin-O staining of the new tissue in the MF and GelMA/ECM groups was less uniform and weaker than that of the GelMA/ECM-PFS group tissue. Similarly, the new tissue in the MF group was negative for toluidine blue staining. Toluidine blue staining was observed in the GelMA/ECM group 3 months after implantation. In the GelMA/ECM-PFS group, toluidine blue staining was observed 3 months after surgery. The repaired tissue in the MF group was negative for type II collagen staining, and the GelMA/ECM group was only partially positive. However, the GelMA/ECM-PFS group was strongly positive for type II collagen.

The histological evaluation of the GelMA/ECM-PFS group at 6 months is shown in Fig. 7A. H&E staining revealed that the structure of the repaired tissue in the GelMA/ECM-PFS group was more lacquered and contained more cells than that in the MF and GelMA/ECM groups. Safranin-O staining showed that the cartilage-specific ECM in the repaired tissues of the GelMA/ECM-PFS group had a homogeneous intensity and was obviously integrated. ECM Safranin-O staining was not noticeable in most of the repaired tissues in the MF and GelMA/ECM groups. Toluidine blue staining of the repaired tissue in the GelMA/ECM-PFS group was almost the same as that of the natural cartilage, but the cartilage was slightly thinner. Most of the repaired tissues in the MF and GelMA/ECM groups were negative for toluidine blue staining. At 6 months postoperatively, the repaired tissue treated with GelMA/ECM-PFS hydrogel showed strong type II collagen staining. The MF group was negative for type II collagen staining. There was very little type II collagen staining in the GelMA/ECM group.

In general, as the repair process progressed, the histological scores of all stent groups gradually increased, and the ICRS scores of repaired tissues in the GelMA/ECM-PFS group were significantly improved compared with those of the MF and GelMA/ECM groups 3 and 6 months after implantation (Fig. 7B, C). Overall, more positive staining of the cell filling, cartilaginous ECM, and collagen type II was observed in the GelMA/ECM-PFS group than in the other groups, indicating that GelMA/ECM-PFS enhances hyaline cartilage regeneration in vivo.

Moreover, we used the Hydroxyproline Assay Kit and the Tissue Total GAG Content DMMB Colorimetry Kit to evaluate total collagen and GAG contents. At both time points, the amount of total collagen and GAG produced in the GelMA/ECM-PFS group was higher than that produced in the other groups (GelMA/ECM group and control group), which was consistent with the above results (Fig. 7D, E).

## Discussion

Promoting the recruitment of endogenous MSCs for the in situ reconstruction of damaged tissues has been proven to have good therapeutic effects and is an alternative method for traditional cell transplantation and tissue regeneration techniques [26]. The strategy is convenient and cost-effective, non-immunogenic, and does not require stem cell culture in vitro or implantation in vivo [27]. Instead, the approach relies on autologous host cells. Therefore, the construction of ideal biomaterials to generate an appropriate artificial cell microenvironment for the recruitment of somatic cells and regulation of recruited stem cell and local tissue-specific cell function is a key issue [28]. The microenvironment of hydrogel materials is highly hydrated, similar to that of natural ECM, allowing efficient transfer of various solutes and nutrients [29]. In addition, hydrogels often have biocompatible and/or biodegradable structures that can deliver bioactive molecules to the target site and promote cartilage regeneration [12].

In our study, we selected ECM and GelMA to construct a simple and convenient crosslinking method to construct bionic cartilage ECM hydrogel materials. Due to their excellent biocompatibility, adjustable mechanical properties, low immunity and abundant availability, gelatin-based materials have been widely used in biomaterial development [30]. It has been reported that GelMA, as an injectable collagen-based biomaterial, may be an ideal material for cartilage repair [14]. ECM is derived from natural cartilage and generally lacks cells and residual cellular components [31]. Compared with natural cartilage, ECM has lower antigenicity and better biocompatibility [31]. Both in vitro and in vivo studies have revealed that chondrogenic stem cells differentiate well in ECM and that ECM improves the efficacy of defect repair [16]. However, as ECM homogenates lack mechanical strength, we generated a structural matrix for cartilage tissue engineering by mixing ECM homogenates of acellular cartilage and GelMA and crosslinking this composite with blue light irradiation after in situ injection to fill cartilage defects.

After constructing the optimized combination of hydrogels, it is necessary to further modify the homing ability of mesenchymal stem cells to the articular cavity by incorporating biochemical signals that recruit stem cells [23]. In this study, we modified the ECM with the verified bone marrow homing peptide PFS for the first time and added PFS-ECM particles to the GelMA hydrogel to construct a tissue-engineered hydrogel scaffold with a specific MSC recruitment function to promote the homing of MSCs in vivo and in vitro (Fig. 1). Fluorescence images confirmed the successful modification of rhodamine-labeled PFS on ECM sponges (Additional file 1: Fig. S1). SEM results showed that the GelMA/ECM-PFS hydrogel had a similar three-dimensional porous structure and higher porosity (> 70%) (Fig. 2D, E), indicating that the addition of ECM-PFS particles did not alter the original bionic structure of the hydrogel. The GelMA hydrogel macro pores were approximately 150–200 μm, and the GelMA/ECM-PFS hydrogel micro pores were 80–100 μm (Fig. 2F). These pore sizes can promote MSC adhesion, proliferation and ECM formation [28]. Moreover, the composite hydrogels have also been shown to have good swelling properties and appropriate degradation (Fig. 2G, H).

Then, BMSCs derived from rabbits were embedded in hydrogel to construct tissue-engineered cartilage in vitro, and the active morphology and biological characteristics of the cells were tested. Live/dead staining, a CCK-8 assay and cytoskeleton staining (Fig. 3) showed that the GelMA/ECM-PFS composite hydrogel not only supported cell growth and proliferation but also promoted cell expansion and diffusion. This may be due to the three-dimensional pore structure provided by hydrogels, and previous studies have shown that enhancing the ability of cells to spread out and connect to neighboring cells in the three-dimensional space of biomaterials is critical for remodeling new cartilage into native tissue [32, 33]. Additionally, naturally occurring cell binding and protease cutting molecules (such as adhesion peptide RGD) in the ECM also play a role [34]. In summary, PFS-modified ECM has affinity for BMSCs, which can greatly improve BMSC homing, adhesion and proliferation.

In addition to providing a good growth microenvironment, the scaffold system should have the ability to effectively recruit MSCs, which is critical for successful tissue regeneration. In vitro Transwell cell migration experiments (Fig. 4A, B) showed that ECM can recruit cells to a certain extent due to its hyaluronic acid content, while PFS peptides in functional hydrogels can quickly and effectively attract additional BMSCs. Moreover, to determine the ability of the three hydrogels to recruit BMSCs in vivo, a rat full-thickness cartilage defect model combined with MF surgery was established. At 2 weeks after in vivo hydrogel implantation, immunofluorescence results showed that the total cell number per region of interest was higher in the GelMA/ECM and GelMA/ECM-PFS composite hydrogels, with a more uniform and abundant cell distribution (Fig. 4C, D). Furthermore, the increase in migrated endogenous MSCs within the ECM-PFS-containing scaffold was demonstrated by the significantly higher number of CD90 and CD105 double-positive cells (Fig. 4C, E). These results collectively suggested that the PFS component can effectively promote the capability of the scaffolding system to recruit BMSCs to the defect site. Since PFS functionally imitates bone marrow homing peptides, MSCs recruited by PFS are likely to originate from the bone marrow. In addition, we speculate that cells from the synovium, adipose tissue and even vascular tissue in the joint cavity may also be mobilized and participate in the regulation of cartilage repair and reconstruction [35, 36]. However, the specific types and mechanisms of action of these MSCs remain unclear, and the origin of these cells and their contribution to tissue regeneration need to be further studied [37].

To explore the chondrogenic effects of composite hydrogels, chondrogenic experiments in vitro were conducted. After culturing tissue-engineered cartilage in chondrogenic differentiation medium, RT-qPCR (Fig. 5A–C) revealed that the expression level of the cartilage-specific marker gene COL2A1 in BMSCs in the GelMA/ECM-PFS hydrogel group was significantly higher than that in the other groups after 14 days. Thus, it was proven that the GelMA/ECM-PFS hydrogel had a strong ability to induce BMSC chondrogenic differentiation. Since the microenvironments of stem cells from different tissues are different, biomaterials with bionic tissue-specific microenvironments promote the directional differentiation of stem cells [38]. The addition of ECM to GelMA hydrogel supplemented specific extracellular matrix components, generating a hydrogel with a similar composition to natural cartilage ECM and providing cells with a microenvironment closer to that of cartilage tissue, thus promoting the differentiation of MSCs into chondrocytes. In addition, ECM may retain some cytokines or structural proteins that influence the differentiation of MSCs into cartilage [39]. The hydrogel with ECM-PFS was confirmed to further promote the differentiation of BMSCs into cartilage, possibly because the addition of PFS changed the attachment state and morphology of the BMSCs in the hydrogel, promoting the secretion of more extracellular chondrocyte matrix components into the microenvironment, generating an environment close to that of natural cartilage [34]. The specific mechanisms underlying the effect of ECM-PFS need to be further studied.

In conclusion, the functional hydrogel GelMA/ECM-PFS not only provides a microenvironment conducive to stem cell cartilage differentiation but also rapidly recruits more endogenous stem cells. We believe that the functional hydrogel GelMA/ECM-PFS is a promising biological material for cartilage tissue engineering. To further confirm the effect of functional hydrogels on cartilage repair, male New Zealand white rabbits were selected as an animal model. Histological examination 3 and 6 months after implantation revealed that GelMA/ECM-PFS was more effective than GelMA/ECM or MF for promoting cartilage repair, further confirmed by macroscopy, micro-CT scanning, histomorphometry, biochemistry and biomechanics (Figs. 6, 7). The results showed that our PFS-functionalized ECM hydrogel scaffold had a satisfactory effect on cartilage regeneration and successfully protected articular cartilage from further damage. Although GE/PFS repaired cartilage damage, the resulting cartilage was still different from normal cartilage. Obtaining repair closer to normal cartilage may require the modification of the hydrogel with more biochemical and biophysical regulatory signals to help complete cartilage regeneration. In addition, the molecular and cellular mechanisms of stem cell involvement in cartilage injury defects are not fully understood. Therefore, further studies are needed to answer these questions.

## Conclusion

This study developed a target-specific hydrogel system by combining a functionalized PFS-ECM particle and GelMA hydrogel for cartilage repair. The ECM-PFS particles enhanced the biomechanical properties of this hydrogel scaffold system, creating a microenvironment for chondrogenic differentiation and BMSC recruitment, while GelMA provided a favorable three-dimensional cell-supporting microenvironment that could bind tightly to host cartilage and fill defects. In addition, the dual-function scaffold showed good cartilage repair effects in rabbit models. The synergy between cell homing and cartilage differentiation processes may provide a promising strategy for cartilage tissue engineering.

## Supplementary Information

**Additional file 1.** Hydrogel composite scaffolds achieve recruitment and chondrogenesis in cartilage tissue engineering applications.
