# Supplementary material for: Hydrogel composite scaffolds achieve recruitment and chondrogenesis in cartilage tissue engineering applications
Source: J Nanobiotechnology. 2022 Jan 6;20:25. doi: 10.1186/s12951-021-01230-7 (PMC8740469; doi:10.1186/s12951-021-01230-7)
Supplement: Supplementary file 1 — Additional file 1. Hydrogel composite scaffolds achieve recruitment and chondrogenesis in cartilage tissue engineering applications. [file 12951_2021_1230_MOESM1_ESM.docx]

Additional Information

Hydrogel composite scaffolds achieve recruitment and chondrogenesis in cartilage tissue engineering applications

Bo Huang^1,2#^, Pinxue Li^1#^, Mingxue Chen^3^, Liqing Peng^1,2^, Xujiang Luo^1^, Guangzhao Tian^1^, Hao Wang^1,2^, Liping Wu^4^, Qinyu Tian^1,2^, Huo Li^1,2^,Yu Yang^1^, Shuangpeng Jiang^1^, Zhen Yang^1^, Kangkang Zha^1^, Xiang Sui^1^, Shuyun Liu^1*^, Quanyi Guo^1*^

1: Institute of Orthopedics, Chinese PLA General Hospital; Beijing Key Lab of Regenerative Medicine in Orthopedics; Key Laboratory of Musculoskeletal Trauma & War Injuries PLA; No.28 Fuxing Road, Haidian District, Beijing 100853, People’s Republic of China.

2: Department of Bone and Joint Surgery, The Affiliated Hospital of Southwest Medical University, No. 25 Taiping Road, Jiangyang District, Luzhou, Sichuan, 646000, People’s Republic of China.

3: Department of Orthopaedics, Beijing Jishuitan Hospital, Beijing 100035, China.

4: Hebei Medical University, Shijiazhuang 050017, Hebei Province, China.

#These authors contributed equally to this work.

*Corresponding author:

(1) Quanyi Guo, Institute of Orthopedics, Chinese PLA General Hospital; Beijing Key Laboratory of Regenerative Medicine in Orthopedics; Key Laboratory of Musculoskeletal Trauma & War Injuries PLA; 28 Fuxing Road, Haidian District, Beijing 100853, People’s Republic of China.

Phone Number: 86-10-6693-6637

Email: doctorguo_301@163.com

(2) Shuyun Liu, Institute of Orthopedics, Chinese PLA General Hospital; Beijing Key Laboratory of Regenerative Medicine in Orthopedics; Key Laboratory of Musculoskeletal Trauma & War Injuries PLA; 28 Fuxing Road, Haidian District, Beijing 100853, People’s Republic of China.

Phone Number: 86-10-6693-6637

Email: clear_ann@163.com

**Additional Materials and Methods**

1.1 Preparation of GelMA and ECM

The preparation of GelMA was based on a previous method[1, 2], with minor modifications. In brief, Type A gelatin was dissolved in PBS at a concentration of 10% (w/v). The mixture was heated to 50°C and stirred continuously until it became transparent (approximately 60 minutes). Methacrylic anhydride (MA) (0.6 g MA per 1 g gelatin) was added slowly, and the mixture was stirred at 50°C for another 60 minutes. The mixture remained homogeneous and opaque. Then, the mixed solution was transferred to a 50-mL centrifuge tube and centrifuged at 3500 g for 3 minutes. The supernatant was removed and diluted 5 times in warm ultrapure water and transferred to a dialysis bag (molecular weight cutoff: 12 kDa). The sample was dialyzed at 40°C for 7 days, and the dialysis water was changed twice a day. After lyophilization, a GelMA macromolecular solid was prepared and stored at -20°C.

As previously mentioned[3], ECM was prepared using physicochemical methods from fresh porcine articular cartilage tissue with some modifications, and our research team previously validated the ECM composition[4]. Briefly, sections of femoral condyle and femoral head cartilage were collected from pigs, washed with PBS, and sterilized with acidic oxidized potential water for 30 minutes. The washing and sterilization process was repeated 3 times. The cartilage pieces were minced and put into a grinder; distilled water was added, and the pieces were repeatedly crushed at 4°C to obtain a suspension of cartilage slurry. The cartilage slurry was diluted 10X with sterile triple-distilled water, frozen at -20°C under hypotonic conditions, and thawed at room temperature. Repeated freezing and thawing of samples 5-6 times resulted in cell inactivation and rupture. The remaining cells in the cartilage slurry were removed by gradient centrifugation at 1500, 3000, and 6000 revolutions per minute in a low-temperature high-speed centrifuge for 20 minutes. The supernatant was collected after centrifugation at each speed. Finally, the slurry was centrifuged at 10,000 revolutions/minute for 30 minutes 3 times to obtain a decellularized cartilage microfilament slurry. The ECM homogenate (3%, w/v) was prepared by PBS dilution and stored at 4°C for subsequent experiments.

To construct a GE composite hydrogel, four different concentrations of (0, 1, 1.5 and 2% w/v) CECM were added to the GelMA solution (10%, w/v), and the concentration of LAP photoinitiator was 0.25% w/v. Then, 500 μL of the prepared hydrogel precursor solution was injected into a cylindrical mold with a diameter of 1 cm, and the GE composite hydrogel was formed after 2 minutes of blue light (405 nm) irradiation.

1.2 GelMA and GE hydrogel compression tests

To determine the ECM concentration resulting in GE composite hydrogels with the best mechanical properties, after the height and diameter of the cylindrical composite hydrogel were measured, samples from the four hydrogel groups (n=3) were fixed on the stage of an Instron 5969 mechanical analyzer. All samples were prepressed at 0.06 N before recording and subsequently pressed at a constant rate of 2 mm/min until fragmentation. The initial size, displacement and applied force were recorded during the compression process. The stress–strain curve was drawn after the completion of the test, and the compression modulus of each sample was calculated according to the 10% - 20% strain interval.

1.3 Construction of ECM/PFS and GelMA/ECM-PFS

To increase the adhesion and viability of BMSCs in the ECM and the ability of composite hydrogels to recruit endogenous stem cells, we designed and prepared PFS-functionalized ECM and added it to a GelMA hydrogel. In short, an ECM homogenate (3% w/v) suspension in a cylindrical mold (3.5 mm in diameter; 20 mm in height) was frozen overnight at -80 ℃ and freeze-dried for 24 h to obtain a CECM sponge column. CECM sponges were soaked in 5 mL of morphine ethyl sulfonic acid (MES, 0.1 M, pH = 6) for 30 minutes, and then 20 mg of 1-ethyl-3-[3-dimethylaminopropyl] carbodiimide hydrochloride (EDC, J&K) and 30 mg of N-hydroxysuccinimide (NHS, J&K) were added for 20 minutes to activate the carboxyl groups of the CECM sponge. The activation buffer was then removed, and the CECM sponge was rinsed three times with sterile PBS buffer 3 times. PFS (500 μg) was added to 5 mL of PBS and reacted in a reciprocating vibrator for 12 h at room temperature. Then, the PFS-functionalized DCECM sponge was rinsed with PBS 3 times to remove the residual chemical reagents. After ultrasound treatment using a Q125 Sonicator (Qsonica, USA), an ECM composite hydrogel consisting of ECM (1%, w/v), GelMA (10%, w/v) and LAP (0.25%, w/v) was prepared. After thorough mixing, 500 μL of the mixture was transferred to the same cylindrical mold and exposed to blue light (405 nm) for 2 minutes to prepare a PFS-functionalized hydrogel. Rhodamine B-labeled PFS (RhoB-PFS) was synthesized to observe the density of BMHP1 conjugated to the ECM sponge by linking carboxylic rhodamine B (Sigma Aldrich, USA) to the N-terminus of PFS. The RhoB-labeled PFS-functionalized ECM sponge was imaged using a confocal microscope to evaluate the PFS distribution in the ECM sponge.

The biologically active functional peptide fragment PFS (amino acid sequence: Ac-GGSKPPGTSS-CONH2) of bone marrow homing peptide (BMHP) used in this study was synthesized by Shanghai Qiangyao Biotechnology Co., Ltd. with solid-phase synthesis technology. Before delivery, the peptide purity was confirmed to be over 95% by high-performance liquid chromatography (HPLC).

1.4 Characterization of composite hydrogels

1.4.1 Morphological characterization

The morphology of the hydrogels was observed using a stereomicroscope (SMZ2, Nikon, Japan), and the microstructure of the scaffolds was further characterized by SEM (S-4800 field emission scanning electron microscope; Hitachi, Tokyo, Japan). Samples were prepared by cutting three freeze-dried hydrogels, spraying the cut gels with gold and placing them under SEM to observe and collect images.

1.4.2 Pore size and porosity measurement

The pore size of the lyophilized hydrogels was observed by SEM, and the mean pore size was calculated by ImageJ software. The porosity of the scaffolds was measured using the ethanol displacement method. V1 represents the initial ethanol volume in a cylinder, and V2 represents the total volume of the scaffolds and ethanol after the scaffold pores were completely filled with ethanol. The residual ethanol volume (V3) in the cylinder after the scaffolds were removed was also measured. The porosity was calculated according to the following equation: Porosity= [(V1-V3)/(V2-V3)] ×100%.

1.4.3 Swelling properties of hydrogel

To determine the changes of ECM particles and ECM-PFS particles on the swelling properties of GelMA hydrogel. The original mass (W0) of freeze-dried hydrogels in each group was weighed and recorded. Then, the samples were placed in PBS solution at 37°C and removed at predetermined time points (1 h, 2 h, 4 h, 6 h, 10 h and 24 h). The water on the surface of the hydrogels was removed with filter paper, and the hydrogels were reweighed to calculate their mass (Wt). The mass swelling ratio of the hydrogels was calculated according to the following formula:

λ(w)=(Wt-W0) ∕ W0. The swelling ratio was expressed as the mass ratio to reach swelling equilibrium.

1.4.4 In vitro enzymatic degradation

As previously mentioned[5], the stability of hydrogels was determined by analyzing the effect of enzymatic degradation using type II collagenase. Freeze-dried and preweighed samples were prepared and incubated for 4 h in an enzyme solution containing 10 U/mL type II collagenase in PBS. The residual weight was subsequently measured hourly, and the degree of degradation was calculated according to the following formula:

Remaining weight (%) = (𝑊_0_−𝑊_𝑑_)/𝑊_𝑑_×100, where 𝑊_0_ is the original dry weight and 𝑊_𝑑_ is the dry weight after incubation for a period of time.

1.5 Cell isolation and culture

The study was approved by the Ethical Committee of PLA. BMSCs were extracted from the bone marrow of New Zealand white rabbits by Percoll density gradient centrifugation, as previously reported[6, 7]. Briefly, the cells were resuspended in Dulbecco's modified Eagle's medium (DMEM, Corning)/F12 containing 10% fetal bovine serum and transferred to 25-cm^2^ flasks (5% CO_2_, 37°C). The medium was carefully replaced three days later, and the nonadherent cells were poured out to obtain adherent BMSCs. Passage 3 (P3) BMSCs were used in subsequent experiments.

We proved the adipogenic, osteogenic and chondrogenic differentiation potential of BMSCs through a trilineage-induced differentiation experiment. BMSCs at passage 2 were used in subsequent experiments. For the adipogenic experiment, 1X10^5^ BMSCs were cultured in a 6-well plate and MSC adipogenic differentiation medium (Cyagen Biosciences, Guangzhou, China). After 7 days of culture, the BMSCs were fixed with paraformaldehyde, and the degree of adipogenesis was determined by Oil red O staining. The osteogenic induction culture method was similar to that used for adipogenic differentiation; however, the BMSCs were cultured in osteogenic differentiation medium (Cyagen Biosciences, Guangzhou) for 14 days and stained with Alizarin red to visualize chondrogenic differentiation. BMSCs (3X10^5^) were centrifuged at 250 g for 5 min in 15-mL Falcon tubes to form cell pellets. The pellets were maintained at 37°C with 5% CO_2_ in basal media for 24 h, and each pellet was nourished with chondrogenic differentiation medium (MSCgo^TM^, Biological Industries, Israel) that was replenished every 3 days for 21 days. Finally, frozen sections were prepared from the pellets and stained with Alcian blue.

A suspension of 1 × 10^6^ (1 mL) was placed in a centrifuge tube, and BMSCs were identified using flow cytometry (Beckman Coulter, CytoFLEX). The antibodies for positive surface markers included CD 90-APC (BD Biosciences, 561409) and CD105-PE/Cy7 (NB500-452PECY7), while the negative markers included CD 34-FITC (Novus, NB2-54355F) and CD 45RA-APC/Cy7 (BD Biosciences, 561624).

1.6 In vitro cytocompatibility study

1.6.1 Live/Dead staining

To assess the viability of BMSCs in different hydrogels, a Live/Dead Assay Kit (Beyotime, Shanghai, China) was used. In short, after digestion and centrifugation, P3 BMSCs were resuspended in the precursor solution of the three hydrogels at 15×10^6^ cells/mL. The hydrogel was formed by irradiation with blue light and cultured with DMEM containing 10% FBS for 1 and 7 days. After culture, the hydrogel-cell complex was added to 200 μL of staining solution containing 1 mM calcein AM and 2 mM ethidium homodimer-1 and incubated in a dark room at 37°C. Finally, images were obtained under a confocal fluorescence microscope (Nikon, Japan) after rinsing with PBS.

1.6.2 CCK-8 assay

Cell Counting Kit-8 (CCK-8; Dojindo, Japan) was used to quantify the proliferation rate of BMSCs in different hydrogels. For the CCK-8 assays, BMSCs were inoculated into each hydrogel scaffold at a density of 5000 cells per well by adding 100 μL of hydrogel precursor solution to each well in a 96-well plate and cross-linking with blue light. After coculturing for 1, 4 and 7 d, the culture media was refreshed with 110 μL of working solution (CCK-8 reagent/cell culture media = volume ratio of 1:10) and incubated at 37°C for 2 h. Then, the optical density (OD) of the CCK-8 solution at 450 nm was measured with a microplate analyzer (Beckman, Fullerton, CA, USA).

1.6.3 Morphology of BMSCs in hydrogels

The morphology of BMSCs in GelMA, GelMA/ECM and GelMA/ECM-PFS hydrogels was assessed by staining with 4',6-diamidino-2-phenylindole (1:100, Life Technologies, CA, USA) and FITC-phalloidin (Cytoskeleton, USA) after 1 d and 7 d of culture according to the manufacturer’s instructions. After the hydrogel-cell composites were washed 3 times with PBS, images were collected using a confocal fluorescence microscope.

1.6.4 Morphology of BMSCs on hydrogels

To further explore the affinity of hydrogels to BMSCs in each group, BMSCs (1 × 10^6^) were inoculated on the surface of the aforementioned cell-free hydrogels. After 7 days of culture, the cytoskeleton and nucleus were stained by the above methods and observed under a confocal microscope.

1.7 In vitro and in vivo MSC recruitment

1.7.1 In vitro cell migration assay

To determine the BMSC recruitment ability of composite hydrogels, a Transwell system (Corning, USA) was used. An appropriate amount of DMEM/F12 medium was added to the upper and lower chambers of the Transwell culture plate, and the plate was incubated at 37°C for 24 h before the medium was removed. Hydrogel extracts were prepared by soaking hydrogels in 1 mL of DMEM/F12 medium at 37°C for 24 h in a 12-well plate. The hydrogel extracts were added to each well in the lower chamber with 3-well replicates per group, and 200 μL of medium containing 50,000 BMSCs was added to the upper chamber. After 12 h, unmigrated cells were carefully removed from the upper chamber. The lower chamber was washed with PBS, and 4% paraformaldehyde was added, followed by a 30-minute incubation period. The perforated film was removed, placed on a glass slide, stained with 0.1% crystal violet solution for 30 minutes, washed with distilled water 3 times, dried, covered with neutral resin, and observed and photographed under a microscope. Three 200× fields of view were randomly selected from each chamber. ImageJ software was used to calculate the number of migrated BMSCs.

1.7.2 In vivo endogenous MSC recruitment

To determine the ability of the three hydrogels to recruit BMSCs in vivo, we established a rat full-thickness cartilage defect model combined with MF surgery to investigate the ability of GelMA, GelMA/ECM and GelMA/ECM-PFS hydrogels to enhance BMSC migration. Six male Sprague–Dawley rats (18 weeks old, 200-220 g) were randomly divided into the above 3 groups, and the surgical procedure was the same as described in Section 2.6.1. After 2 weeks, all rats were sacrificed, and the regenerated tissue in the damaged area was sampled. CD90 and CD105 were defined as MSC-specific markers, and the effects of hydrogel recruitment on BMSCs in vivo were determined by immunofluorescence staining. Briefly, the regenerated tissues were fixed with 4% paraformaldehyde for 30 minutes, and Triton X-100 (0.5%) was used to permeabilize samples for 30 minutes. The samples were blocked with immune blocking solution (Beyotime, Shanghai) after washing with PBS. The samples were then incubated overnight at 4°C with primary antibodies against CD90 (1:300, Novus Biologics) and CD105 (1:300, Novus Biologics). After washing, the samples were incubated with secondary antibodies conjugated with Alexa Fluor488 and Fluor594 (1:200, Abcam, UK) for 1 h and DAPI (1:100, Life Technologies, CA, USA) for 10 minutes. Finally, CD90- and CD105-positive cells in the regenerated region were observed by confocal fluorescence microscopy.

1.8 In vitro chondrogenic differentiation

1.8.1 RT–qPCR for cartilage-specific gene expression analysis

To explore whether the incorporation of ECM and ECM-PFS could promote the chondrogenic differentiation of BMSCs in the GelMA hydrogel, we cultured the three hydrogels with BMSCs in chondrogenic differentiation medium (MSCgoTM, Biological Industries, Israel) to construct tissue-engineered cartilage in vitro. After 7 and 14 days of culture, the expression of cartilage-specific genes in BMSCs was measured by RT–qPCR; type II collagen (COL2) was measured to evaluate chondrogenesis, type I collagen (COL1) was measured to evaluate osteogenesis, and type X collagen (COL X) was measured to assess whether BMSCs are prone to hypertrophy. The primer sequences used in our study are shown in Table S1. Total RNA was extracted from BMSCs with a Cell Total RNA Isolation Kit (Foregene, Chengdu, China), and total RNA was converted to complementary DNA using 5 × RT Master Mix (Toyobo, Osaka, Japan). RT–qPCR was performed on a StepOneTM Real-Time PCR system (Applied Biosystems, USA) using a standardization procedure. To verify the reliability of the primers, we established fusion curves for each reaction system, and there was no nonspecific amplification in the dissolution curves. Relative mRNA expression was normalized to that of the housekeeping gene (GAPDH) and calculated using the 2^-ΔΔ^CT method.

1.8.2 Histological and immunohistochemical staining analysis

Tissue-engineered cartilage samples were collected after 2 weeks of culture, fixed with paraformaldehyde, dehydrated, embedded in paraffin, and sectioned at a thickness of 6 μm. After dehydration, sections were stained with Safranin-O (Solarbio, Beijing, China) according to the standard protocol. Type II collagen in tissue-engineered cartilage was evaluated immunohistochemically. In brief, after dewaxing and washing the sections, endogenous peroxidase was removed with hydrogen peroxide. After washing the sections with PBS and sealing, the anti-type II collagen primary antibody (1:200, DSHB, IA, USA) was added, and the sections were incubated at 4°C overnight. Subsequently, secondary antibody was added. After color development with a chromogenic agent, images were collected using a bright field microscope.

1.8.3 Glycosaminoglycan and hydroxyproline analysis

BMSC DNA was extracted by a TIANamp genomic DNA kit (Tiangen, China) and quantified using the Quant-it ™ PicoGreen® dsDNA Reagent and Kit (Invitrogen, USA). The DNA solution was mixed with the fluorescent reagent per standard procedures. The fluorescence of the samples at 480 nm was measured using a fluorescence microplate reader, and the DNA concentration of the samples was calculated according to a standard curve and the weight of the sample. The glycosaminoglycan (GAG) and hydroxyproline (HYP) contents of tissue-engineered cartilage were determined by the Hydroxyproline Assay Kit and the Tissue Total GAG Content DMMB Colorimetry Kit as per the experimental protocol. To prevent GAG and collagen in the ECM from affecting the experiment, we generated a GelMA/ECM hydrogel without BMSCs and measured the GAG and HAP contents as a blank control.

1.9 In vivo animal studies

1.9.1 Surgical procedures

Animal experiments were approved by the Institutional Animal Care and Use Committee at the PLA General Hospital. Thirty-two male New Zealand rabbits (6 months old, 2.5-3.0 kg, n=8 knees per group) were prepared and randomly divided into (4) groups: the microfracture (MF), microfracture combined with GelMA/ECM implantation (GelMA/ECM), microfracture combined with GelMA/ECM-PFS implantation (GelMA/ECM-PFS) and sham groups. The rabbits were disinfected, and then a medial parapatellar approach was used to open the joint cavity. A sterile corneal trephine punch was used to drill a cylindrical hole to create a cartilage defect (diameter 3.5 mm × depth 1.5 mm) in both lower limbs at the center of the groove. After microfracture surgery, each hydrogel precursor solution was injected into the defect area through a syringe. After the liquid level was flush with the surrounding cartilage surface, the area was irradiated with blue light for 2 minutes to form a hydrogel scaffold matching the shape of the defect area. After confirming that the hydrogel was firmly implanted into the defect, the capsule and skin were sutured layer-by-layer. In the MF group, only microfracture surgery was performed, while in the sham group, the joint cavity was opened and sutured without surgery. Knee joints were collected from each group for further evaluation after euthanasia at either 3 or 6 months after surgery.

1.9.2 Macroscopic evaluation and ICRS score

The rabbits were euthanized 3 and 6 months after surgery. The knee cavity was opened at the suture location to observe whether the joint fluid was abnormal and whether there were infections and other abnormalities in the joint. General images were collected with a stereomicroscope (SMZ2, Nikon, Japan) 1.5 cm from the medial and lateral femoral condyle line after osteotomy. Femur specimens from each group were evaluated and scored by 3 independent (blind) researchers using the International Society for Cartilage Research (ICRS) scoring system (Table S2).

1.9.3 Micro-CT analysis

We further evaluated the regenerated tissue with a General Electric (GE) eXplorer Locus SP (GE, Boston, MA, USA) by performing micro-CT scans. After macroscopic evaluation, the samples were positioned in the device for scanning. The scans were then reconstructed to form 3D images, and regions of interest (ROIs) were selected in the defect site of each sample. Bone mineral density (BMD) and bone volume fraction (BVF) were statistically analyzed to evaluate the reconstruction of subchondral bone in each group.

1.9.4 Biomechanical analysis

To determine the biomechanical properties of the tissue, we tested the compression modulus of each group of samples. After micro-CT scanning, the sample was placed in the mechanical analysis instrument and pressed with a probe. The forces used and level of displacement were recorded during the compression process. Young’s modulus was calculated using the following formula: E=(F⋅L)/(S⋅ΔL), where F is the applied pressure, S is the indenter area, L is the total cartilage thickness, and the thickness of the cartilage layer was measured by the probe method.

1.9.5 Histology and IHC analysis

After micro-CT scanning, the samples were collected and fixed with 4% paraformaldehyde for 48 h, decalcified with 10% ethylenediamine tetraacetic acid (EDTA) for 7 weeks, dehydrated, embedded in paraffin, and cut into 6-μm sections. Hematoxylin and eosin (H&E), safranin-O, toluidine blue, and Sirius red were used to evaluate the morphology and arrangement of the neotissue and identify GAGs. Type II collagen secretion in the regenerated tissues was evaluated by immunohistochemistry. After staining, the regenerated tissue was imaged and histologically evaluated according to the ICRS histological scoring system (Table S3) (n = 3 per time point).

1.9.6 Biochemical assays

After biomechanical analysis, the HYP and GAG contents of the regenerated tissue were determined by the Hydroxyproline Assay Kit and the Tissue Total GAG Content DMMB Colorimetry Kit according to the manufacturer’s instructions.

1.10 Statistical analysis

All statistical analyses were performed using SPSS V.20.0 (IBM; Armonk, New York, USA). For normally distributed data, one-way analysis of variance (ANOVA) or Student’s t test was used for quantitative data; otherwise, the nonparametric Kruskal–Wallis test for nonnormally distributed data was applied. Statistical significance was set at a two-sided p value of <0.05.

**Additional Figures and Tables**


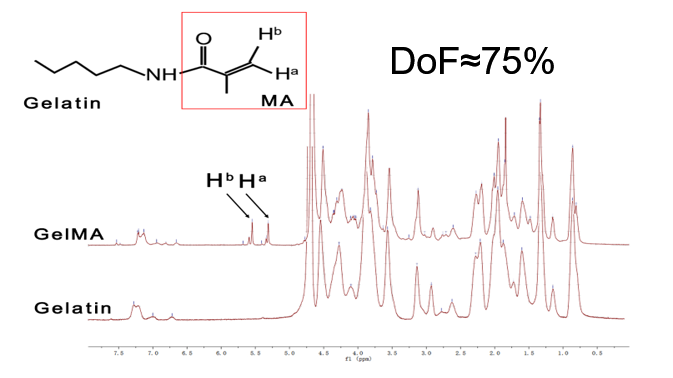


**Figure S1.** The degree of substitution (DS) of GelMA.


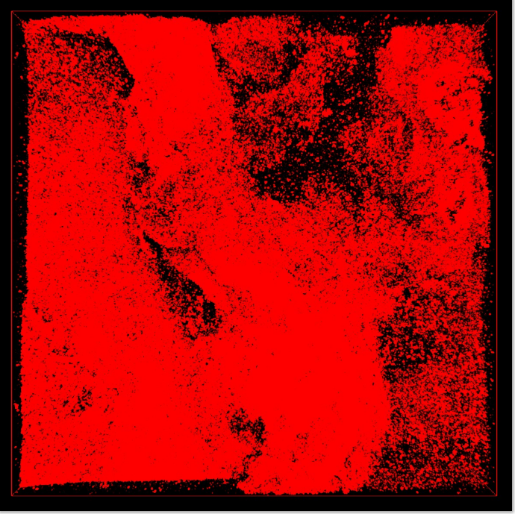


**Figure S2.** Fluorescent image of the ECM sponge (RhoB-PFS).

**
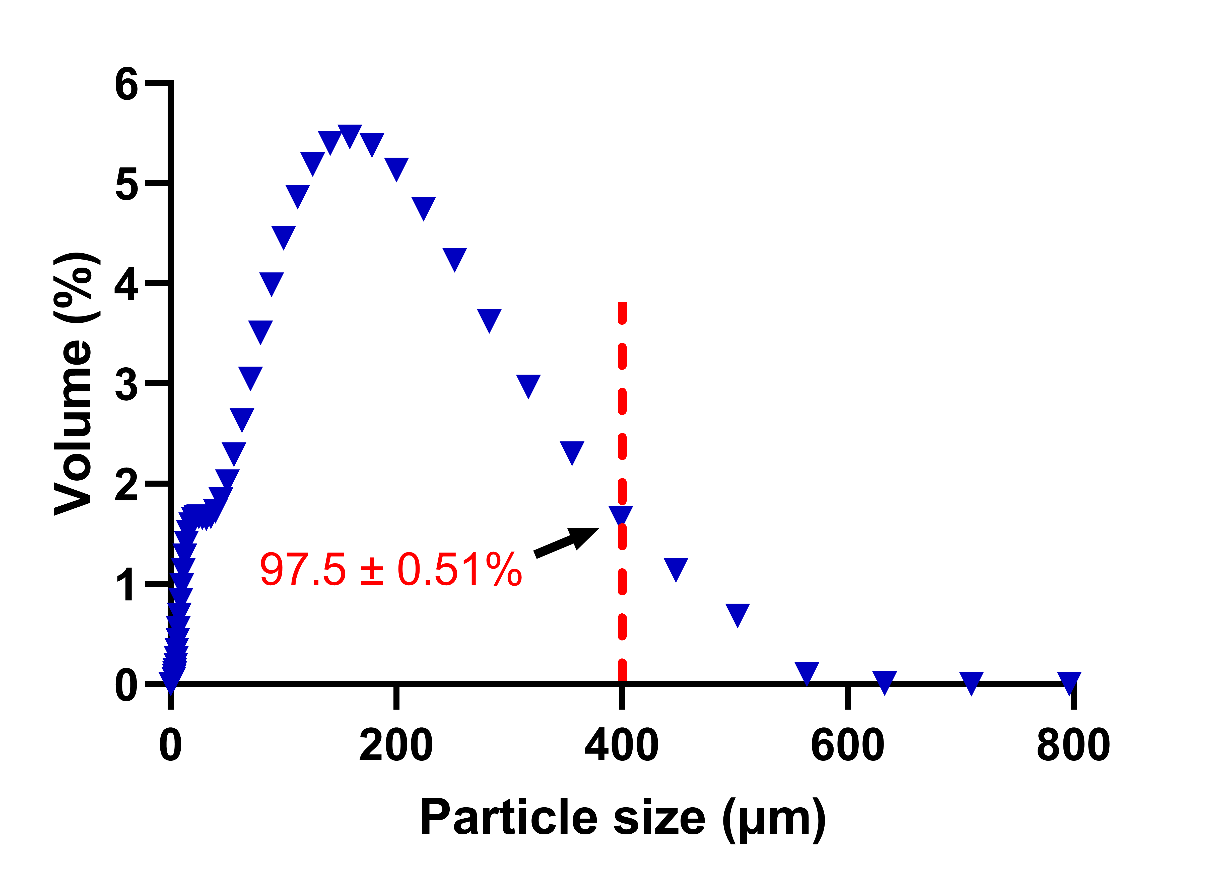
**

**Figure S3.** Quantitative distribution of the PFS-ECM particle size.

**REFERENCES**

[1] D. Loessner, C. Meinert, E. Kaemmerer, L. Martine, K. Yue, P. Levett, T. Klein, F. Melchels, A. Khademhosseini, D. Hutmacher, Functionalization, preparation and use of cell-laden gelatin methacryloyl-based hydrogels as modular tissue culture platforms, Nat Protoc 11(4) (2016) 727-46.

[2] Z. Jian, T. Zhuang, T. Qinyu, P. Liqing, L. Kun, L. Xujiang, W. Diaodiao, Y. Zhen, J. Shuangpeng, S. Xiang, H. Jingxiang, L. Shuyun, H. Libo, T. Peifu, Y. Qi, G. Quanyi, 3D bioprinting of a biomimetic meniscal scaffold for application in tissue engineering, Bioactive materials 6(6) (2021) 1711-1726.

[3] Q. Yang, J. Peng, Q. Guo, J. Huang, L. Zhang, J. Yao, F. Yang, S. Wang, W. Xu, A. Wang, S. Lu, A cartilage ECM-derived 3-D porous acellular matrix scaffold for in vivo cartilage tissue engineering with PKH26-labeled chondrogenic bone marrow-derived mesenchymal stem cells, Biomaterials 29(15) (2008) 2378-87.

[4] G. Tian, S. Jiang, J. Li, F. Wei, X. Li, Y. Ding, Z. Yang, Z. Sun, K. Zha, F. Wang, B. Huang, L. Peng, Q. Wang, Z. Tian, X. Yang, Z. Wang, Q. Guo, W. Guo, S. Liu, Cell-free decellularized cartilage extracellular matrix scaffolds combined with interleukin 4 promote osteochondral repair through immunomodulatory macrophages: In vitro and in vivo preclinical study, Acta biomaterialia 127 (2021) 131-145.

[5] S. Gorgieva, V. Kokol, Preparation, characterization, and in vitro enzymatic degradation of chitosan-gelatine hydrogel scaffolds as potential biomaterials, Journal of biomedical materials research. Part A 100(7) (2012) 1655-67.

[6] J. Ning, C. Li, H. Li, J. Chang, Bone marrow mesenchymal stem cells differentiate into urothelial cells and the implications for reconstructing urinary bladder mucosa, Cytotechnology 63(5) (2011) 531-9.

[7] J.N. Beresford, J.H. Bennett, C. Devlin, P.S. Leboy, M.E. Owen, Evidence for an inverse relationship between the differentiation of adipocytic and osteogenic cells in rat marrow stromal cell cultures, J Cell Sci 102 ( Pt 2) (1992) 341-351.
